# Measles in Canada: modelling outbreaks with variable vaccine coverage and interventions

**DOI:** 10.1186/s12879-025-10564-8

**Published:** 2025-02-19

**Authors:** Jennifer McNichol, Javad Valizadeh, Samara Chaudhury, Caroline Colijn

**Affiliations:** Department of Mathematics, Simon Fraser University, Burnaby, British Columbia Canada

**Keywords:** Measles, Outbreak, Simulation, SEIR, Vaccination

## Abstract

**Background:**

The global incidence of measles has increased markedly since 2023. In Canada, where measles has had elimination status for more than two decades, most cases can typically be traced to travel. While the majority of Canadians are vaccinated against the measles virus, or considered immune due to previous infection, there are communities with low vaccination coverage.

**Methods:**

In this study, we develop a stochastic Susceptible-Exposed-Infectious-Recovered model to explore what measles outbreaks could look like upon importation into Canada under a number of scenarios, vaccination coverage levels, and public health interventions. We collect reports of real-world measles outbreaks and compare them to our model outbreaks’ size and duration.

**Results:**

Our model suggests that community level outbreaks can be controlled at or above 85% vaccination coverage with public health interventions and that above 95% coverage, 99% of measles introductions do not result in an outbreak. Below 85% coverage, outbreaks in small communities (size 1000) with relatively strong public health measures range from median size of under 4 (80% coverage) to 186 (55%), comparable to reported outbreaks in Canada and elsewhere. Outbreaks very often last under 60 days. We characterize how outbreak sizes and durations depend on the strength of interventions, community size and vaccination coverage. We make the model available as a web-based ‘shiny’ application.

**Conclusions:**

Since the vast majority of measles cases in Canada can be traced to imported cases, our model serves as a last step in the chain of actions needed to bridge from global measles outbreaks to local scenarios within Canada. Given cases entering Canada, we are able to project the duration and size of an outbreak, helping to inform the public of the measles-related risk.

**Supplementary Information:**

The online version contains supplementary material available at 10.1186/s12879-025-10564-8.

## Background

By May 2024, more than 56,000 cases of measles were reported to the World Health Organization (WHO) European region, nearly reaching 2023 levels even before halfway through 2024 [1]. This has raised global concern for measles prevention. In Canada, where the majority of the cases in 2024 were either imported or linked to imported cases, and less than 5% of cases were sporadic with an unknown source of exposure, 77 cases of measles were reported as of May 24, 2024 [2]. In contrast, 12 cases were reported in 2023 [3]. A Rapid Risk Assessment issued by the Public Health Agency of Canada (PHAC) in March of 2024 stated that “Canada’s vaccination uptake is below the target of 95% coverage with two doses of a measles vaccine recommended for measles elimination” [4]. Measles has been considered eliminated in Canada since 1998; while high levels of protection in Canadian communities mean that endemic transmission has not been established, communities with low vaccination rates are at risk of outbreaks if introductions do occur. Statistics Canada reported 90% or higher coverage of recommended measles vaccines in the 2017–2021 period, in most places, but there is considerable variability between regions and communities, with some areas, schools or geographies vulnerable to measles outbreaks due to considerably lower vaccination rates [5]. Among the provinces, only New Brunswick, Ontario, and Manitoba require children to be vaccinated against the measles virus prior to school entry [6]; however, exemptions are ubiquitous [7], further contributing to the variation in community coverage. Under-immunized communities are of concern in light of the current rate of measles importation and rises in measles incidence abroad.

**Fig. 1:**
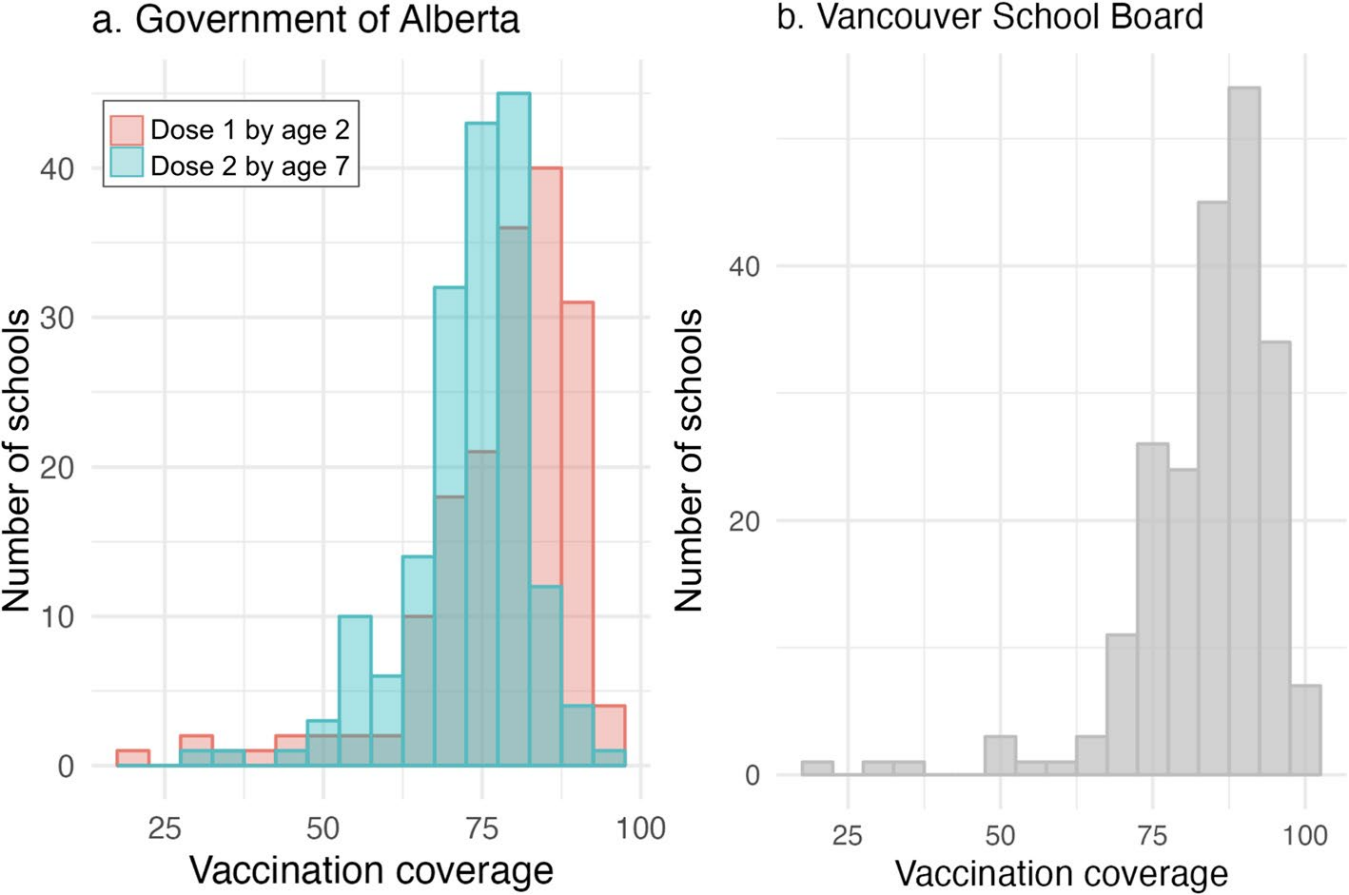
**a** Alberta MMR vaccination coverage by age 2 and age 7 in 2023. **b** Vancouver school board MMR vaccination coverage for children in Kindergarten (approx. age 6) in the 2018/19 school year

For Canadian provinces that do not mandate the MMR vaccine prior to school entry, some data on MMR vaccination coverage is publicly available by age. For example, in Alberta in 2023 [8], MMR coverage can be as low as 32% in some sub-populations but typically ranges from 45 to 95% (see Fig. 1a). Similarly, British Columbia’s regional MMR coverage ranges from 77 to 89% at age 2 and 75 to 92% at age 7, as of 2020 (the most recent year for which data are available). In contrast, Saskatchewan reports very high vaccination levels for measles, with all jurisdictions over 85% coverage and the majority over 90%. In some jurisdictions, school data are also available. For example, Fig. 1b shows Vancouver Coastal Health school vaccination coverage [9], with occasional very low reported rates, and the majority of schools with at least 65% coverage.

Overall, Canadian vaccination for measles ranges from 60% or below in some jurisdictions to above 90% in others, with an overall high average of approximately 90%.

In this study, we use a simple stochastic simulation model to explore how large measles outbreaks in Canada could be and how long they are likely to last. We inform the model with past outbreak sizes in similar populations, well-characterised time frames for the transmissibility of measles and the course of infection, and Canadian heterogeneous vaccination rates.

## Methods

### Model

**Fig. 2:**
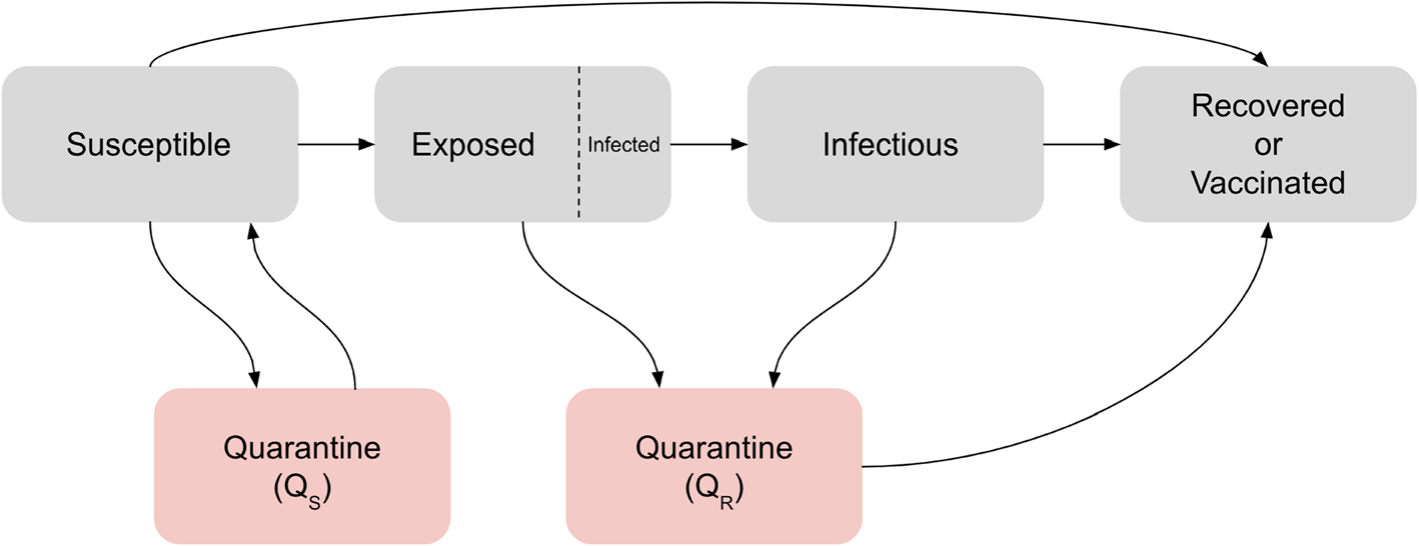
Compartmental model schematic

We model introductions and consequent transmission of measles in communities, using a stochastic susceptible-exposed-infectious-recovered (SEIR) model with additional isolation compartments reflecting isolation of those who are susceptible and potentially exposed, or who have been infected but have not yet become symptomatic. A schematic of this model is given in Fig. 2. The compartments are *S*: susceptible; *E*: “exposed” (i.e. infected with the measles virus) but not yet symptomatic (but some are already infectious prior to the onset of rash); *I*: symptomatic with rash (and infectious); *R*: recovered (immune); $Q_s$: not infected but isolating; $Q_r$: exposed and isolating or having received post-exposure prophylaxis (PEP). We model a fraction of the “exposed” individuals as infectious because there is data on the time to rash onset, we wish the *I* compartment to model individuals with rash, and individuals can be infectious before the onset of rash. This approximates having an additional compartment for those who are infectious but do not yet have rash. Infectious individuals recover at rate $\gamma$ and are identified and isolated at rate $q_i$. Susceptible individuals become infected at rate $\beta (cE + I)S$, where $\beta$ is the transmission parameter and *c* (which is less than one) reflects the fact that individuals become infectious approximately four days before the onset of rash [10] (we explore the case where the *E* class is not infectious and *I* represents all infectious individuals in the Appendix). Susceptible people are isolated at rate $q_s$ (due to knowledge of measles exposures in their community or contacts), and can be vaccinated via an additional public health vaccination effort at rate *v*. Exposed individuals are isolated or given PEP at rate $q_{spep} = q_s + q_{pep}$, or enter the infectious (and transmitting) compartment at rate *k*, reflecting the (inverse of the) duration between infection and rash onset. Recovered individuals remain recovered; while immunity to measles may wane eventually, the waning rate is slow enough that in the $<1$ year time frame of the outbreaks considered here, we do not consider it.

If the model were a deterministic set of ordinary differential equations, it would be written as follows:

$$\begin{aligned} \dot{S} & = -\beta (cE + I)S -vS-q_sS+ \ell Q_s \\ \dot{E} & = \beta (cE + I)S -kE-q_{spep}E \\ \dot{I} & = kE - \gamma I - q_i I \\ \dot{R} & = \gamma I + v S \\ \dot{Q_s} & = q_s S - \ell Q_s \\ \dot{Q_r} & = q_{spep} E + q_i I. \end{aligned}$$

Using the next-generation matrix method we find the basic reproductive number of this model: $R_0 = \frac{c\beta S_0 (\gamma + q_i) + k \beta S_0}{(k+q_{spep})(\gamma + q_i)}$ where $S_0$ refers to the susceptible population at the disease-free equilibrium (*N*).

We simulate a stochastic process with the given transitions and rates using a Gillespie algorithm approach, computing the total rate for all events, sampling an exponential random variable to simulate the time until the next event, and then allocating which event occurs with the appropriate probability. Within each simulation, parameters are fixed; the stochasticity in the model results only from the time to the next event, and the nature of that event.

We use two model populations: 1000, reflecting a school or other small setting with a population in the 100s plus surrounding households and immediate contacts, and 8000, reflecting a close-knit community that is however larger than a very focused school setting. Note that these do not aim to model whole countries, but communities of sizes 1000–8000. Our model settings potentially have a lower-than-average vaccination rate [11]. We initialize our model with two introduced cases in the small population and three in the larger population. This reflects the fact that before cases are being regularly detected, public health interventions and public awareness are unlikely to be heightened, but removes the need to explicitly model a very short early period with reduced detection and isolation (at such a time, it is very likely that an introduced case will lead to another, and in any case, we focus not on the probability of a second or third case, but on the size and duration of outbreaks given a second or third case).

We use reported outbreaks in high-vaccination countries, together with the natural history of measles, to tune model parameters. We validate the model with comparison to past outbreak sizes and durations, though these are variable. We note that at 90% vaccination, measles typically does not spread despite regular introductions and occasional outbreaks. However, at 80% vaccination we would anticipate that substantial public health interventions (additional vaccination, case identification, contact tracing, PEP, isolation of those who are exposed, and isolation of symptomatic individuals) are required to control outbreaks. These interventions are widely used in comparable jurisdictions to Canada [11–13]. Our SEIR model is available as an interactive R Shiny web application: (jmcn.shinyapps.io/measles-canada).

### Validation

Natural history parameters are relatively well-established for measles, but our intervention parameters are difficult to estimate. To ensure that our model creates realistic outbreaks for the kinds of population we consider, we compared model simulations to key features of measles outbreaks: size, duration, and controllability. The values used for our model parameters and rationale are given in Table 1. For $q_i$, the isolation of those with rash, we model that within 0.5 to 1 day of rash onset, most individuals isolate with high effectiveness but this is imperfect (highly transmissible; household contacts; individuals may seek healthcare and have contact there). We use $q_i = 0.4$ per day (1 day x 40%, reflecting both some individuals not isolating and imperfect isolation) in the strong setting, and with less urgent messaging and support for reducing contact, 0.28 per day.

To arrive at the values of $q_s$, and $q_{pep}$, we take into account the time to contact exposed individuals, the number of exposed people who are willing to isolate and the effectiveness of their isolation, as well as the fraction of individuals eligible for PEP, of those people, who accept PEP, and the effectiveness of PEP. Note that these are all rough estimates; more conservative estimates yield weaker interventions. We choose two sets of intervention parameters to explore: “strong” and “weak”. Inspired by the public health response to the March 2024 outbreak in Québec, we assume the time to contact is 2 days in the strong intervention setting and 4 days in the weak setting. Further, we assume 50% of exposed individuals are eligible for PEP in the strong setting (30% for weak) with an acceptance of 90%, and 50% PEP effective in both settings. Some of those who are not eligible for PEP know they were exposed and will isolate. For the fraction of individuals not eligible for PEP we consider the willingness to isolate to be 60% and that this isolation is 50% effective in both strong and weak and settings.

We compute $q_{pep}$, the rate at which infected individuals are removed from risk due to PEP, with: $q_{pep} =$ (1/time to contact) (fraction offered PEP) (fraction who accept PEP if offered)(effectiveness of PEP). With the above numbers this gives 0.1875 per day (strong measures) and 0.0875 (weak measures).

For the rate $q_s$ at which those who have been exposed are removed from risk, suppose it takes 2–3 days to find and contact susceptible people with exposure risk; if half of them reduce contact immediately with approximately 30% effectiveness we have an overall rate $q_s=$ 0.06 per day. If case finding and isolation are less rapid or effective we model this with $q_s=0.04$ per day. In general $q_s$ = (1/ time to contact) ( fraction who agree to reduce contact)(fraction of their contacts removed).

In Minnesota, in a measles outbreak in 2017 [11], just over 1/4 as many people received PEP as were excluded from childcare settings, though the denominators are not given. This suggests that the rates of PEP and isolation/exclusion do not differ by orders of magnitude and that the PEP rate is lower.

**Table 1:** Description of model parameters for strong (first value) and weak (second value) interventions with rationale. Values of rate parameters *c*, *v*, $q_s$, $q_{pep}$, and $q_i$ are per day

Parameter	Value	Description	Reference or rationale
$\beta$	3.75e-4 ($N=1000$); 4.69e-5 ($N=8000$)	Transmission parameter	$R_0$ usual range 12–18; estimate for Canada 28; this gives $\sim 20$ with weak measures and $v=0$ [14].
*c*	0.3	“Exposed” individuals are infectious before onset of rash	4 days infectiousness prior to rash onset [10]
*v*	0.005; 0.003	Supplementary vaccination of susceptibles. Not all jurisdictions strongly encourage additional vaccination in the general population as an outbreak response.	British Columbia vaccination in 2019 outbreak (rough estimate) [15]
$q_s$	0.06; 0.04	Isolation of susceptible individuals following contact tracing, exposure notifications	See text
$q_{pep}$	0.1875; 0.0875	Post-exposure prophylaxis (PEP) plus isolation rate if PEP declined	See text
*k*	1/12	Progression to rash onset	Course of infection rash onset at 10–14 days [10]
$q_i$	0.4; 0.28	Identification and isolation of symptomatic individuals	See text
$\gamma$	1/4	Recovery from symptoms and infectiousness	Infectious until 4 days after rash onset [10]

It is well known that above 95% vaccination measles is not expected to spread, and the consistent experience in Canada since elimination in 1998 (and in similarly highly-vaccinated populations) suggests that even with relatively minor public health interventions, outbreaks are not substantial in size at 90% vaccination coverage and are not large even at 85%. However, once vaccination levels drop below that, at 80% and below, outbreaks are more sizable, and for low vaccination rates, or when public health measures are less intensively focused on case finding, PEP to prevent transmission, and isolation, they can have hundreds of cases [16].

When sizable outbreaks occur in smaller settings and close-knit communities, they tend to last 60–70 days [11, 12, 17] and range in size widely up to approximately 100 cases; the population size is rarely known or reported. Larger outbreaks occur, spreading to larger populations and lasting over longer periods [13, 18].

The WHO defines a measles outbreak as “two or more laboratory-confirmed measles cases that are temporally related (with dates of rash onset occurring 7–21 days apart) and epidemiologically or virologically linked, or both”. To draw a comparison between our model and past real–world outbreaks, we specify here that we consider those outbreaks where an imported case is followed by community spread. In Table A1 we summarize 21 readily available outbreaks world–wide that occurred in comparable regions to those found in Canada, as well as some historic Canadian outbreaks. Note, we did not aim to carry out a systematic literature review, rather, Table A1 serves to place our model results in context. We included outbreak reports that (1) described outbreaks, as opposed to overall measles annual reports for a region (which likely comprise many separate introductions); (2) reported the size of the outbreak, (3) reported the duration; (4) included some information about the the vaccination rate in the community before the outbreak and (5) contained some information about the public health measures in place. We searched google scholar and NCBI. While there is likely some bias in what is reported (with very small outbreaks and introductions that do not lead to outbreaks less likely to be published), this collection of reported outbreaks helps us to compare the model results to real–world outbreaks. Note that our outbreak list is not exhaustive, and not all outbreaks are comparable to our model (in particular, some occur in the general population or in much larger communities than we have modelled).

## Results

### Simulated outbreaks

**Fig. 3:**
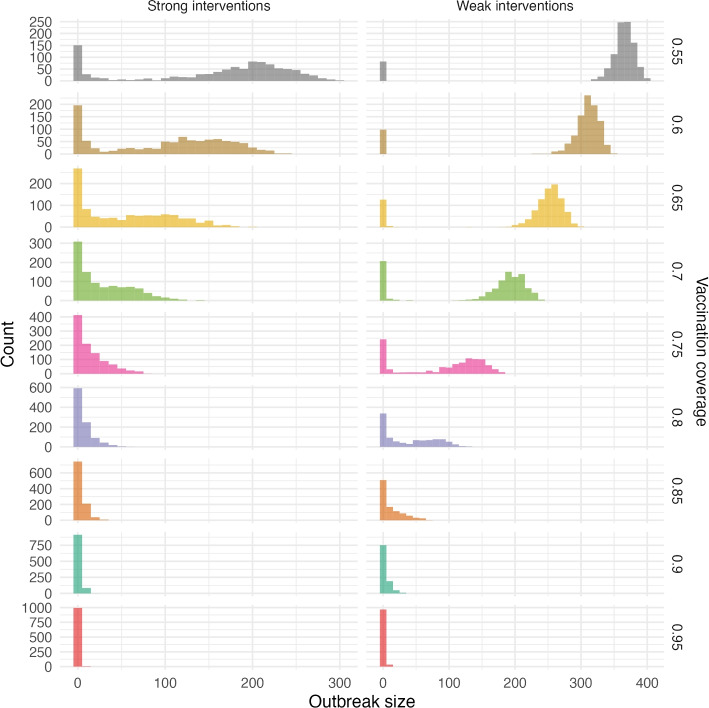
Outbreak sizes for 1000 simulations of a population of size 1000 with strong and weak interventions for various levels of vaccination coverage

Simulated outbreaks from our model in a school-centred population of 1000 are shown in Fig. 3 for both strong and weak levels of public health interventions. The strong interventions control outbreaks relatively well above a vaccination coverage of 0.75 to 0.8, and at those levels, outbreaks can have tens of cases even with interventions in place. Below 70% coverage, the strong interventions are no longer able to control outbreaks and they spread more widely, reaching over 100 individuals and potentially several hundred. With weaker interventions, vaccination coverage below 0.8 will not control outbreaks, as outbreak sizes are frequently over 100 cases even in this small population. Below 70% coverage, weak interventions are not enough to manage outbreaks, leading to outbreak sizes well above 200.

**Fig. 4:**
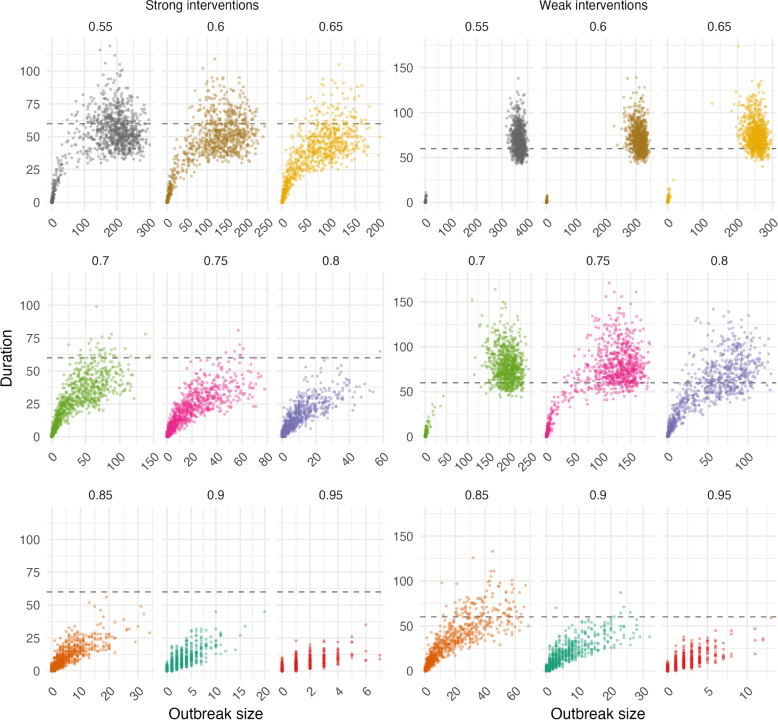
Duration (in days) versus outbreak size over 1000 simulations of a population of size 1000 with strong and weak interventions for various levels of vaccination coverage. Grey dashed line represents 60 days

The duration of an outbreak increases with size, as seen in Fig. 4. With strong interventions, we can usually limit outbreak duration to 60 days with vaccination coverage of 0.80 or higher. In order to limit an outbreak to under 60 days with weak interventions, a small population of 1000 needs to have a vaccination coverage of 0.95.

We found similar results for a population size of 8000. Again, outbreaks can be controlled when the vaccination coverage of the population is at or above 75% and public health interventions are in place (see Fig. A1) and with weaker interventions, a higher vaccination coverage is important. In the larger population size, outbreaks may of course become much larger than in a smaller population; however, the duration of the outbreak does not tend to be much longer. For example, at 85% vaccination coverage and week interventions, our simulated outbreaks in the large population reached a maximum duration of 150 days (Fig. A2), while in the small population the maximum duration was 130 days (Fig. 4). In contrast, outbreaks in the large population reached 175 cases while the outbreak size did not exceed 70 cases in the small population. We report median theoretical values for severe outcomes based on the median outbreak size and known risk rates [19, 20] in Table 2.

**Table 2:** Median outbreak size, and expected numbers of various outcomes at the median outbreak size, given reported risks of these outcomes [19]. We assume complete ascertainment. If some transmission is in undetected cases who acquire immunity, these numbers would be lower. Numbers are overall rates because in the absence of information about the nature of the affected community, we do not model age demographics or contact patterns

Vaccination coverage	Median outbreak size	Hospitalizations	Diarrhea/Pneumonia/Otitis media	Encephalitis/Death
0.95	0	0	0	0
0.9	1	0.2	0.1	0.001
0.85	2	0.4	0.2	0.002
0.8	4	0.8	0.4	0.004
0.75	8.5	1.7	0.85	0.0085
0.7	19	3.8	1.9	0.019
0.65	51	10.2	5.1	0.051
0.6	116	23.2	11.6	0.116
0.55	186	37.2	18.6	0.186

**Fig. 5:**
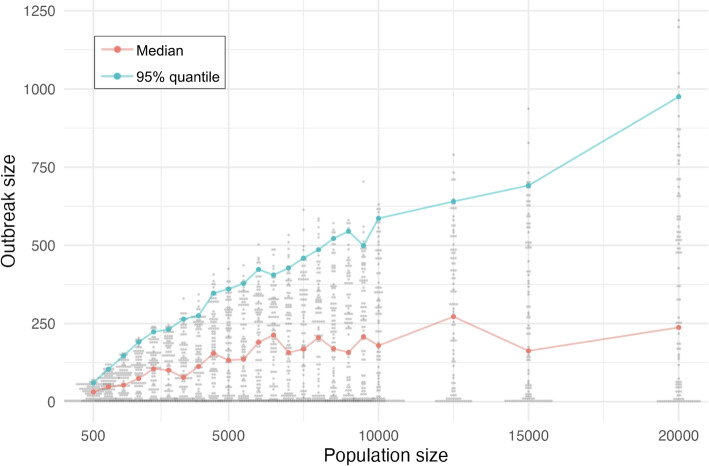
Outbreak size scaling with population size for 80% vaccination coverage in our model with parameter values corresponding to weak interventions. Grey dots depict the distribution of outbreak sizes in each of 100 simulations; the median and 95% quantiles of the simulated outbreak sizes for each population size are overlayed in pink and blue, respectively

We performed sensitivity analysis on the parameters, $q_i$, $q_s$, $q_{pep}$, and *v* by simulating a population size of 1000 with 75% vaccination coverage and varying each parameter independently. The results are provided in Fig. A3. We found that varying the rates that individuals progress into the quarantine compartments, i.e., $q_i$, $q_s$, and $q_{pep}$ yielded a marked increase in outbreak size as these parameters were decreased (independently); a similar but more subtle increase was noted for outbreak duration. Varying *v*, the parameter that accounts for vaccination of susceptible individuals, had no impact on outbreak size nor duration (Fig. A3d). Finally, we carried out 100 simulations of our model under strong interventions with vaccination coverage of 0.8 with varying population size from 500 to 20,000. With this coverage, the outbreaks are “small” in the sense that the mode of the size distribution is 1 (“subcritical”). We found that in this context the median and 0.95 quantiles of the outbreak sizes scale sub-linearly with population size (Fig. 5). In contrast, under lower coverage, the outbreak size will scale linearly with the population size: a modelled community of size 16,000 would have outbreaks twice as large as our outbreaks with population size 8,000. This illustrates how to apply our modelling to communities of different sizes. We conducted 100 simulations to examine the effect of the initial infections imported on outbreak size (Fig. A4). When vaccination coverage is 0.9 we see a moderate increase in outbreak size as the number of initial infections increase but most outbreaks are smaller than the number of initial infections. In contrast at vaccination coverage of 0.7 there is a strong dependence on the number of introductions until 8 initial cases, after which a large outbreak is very likely.

We compared model simulations to real reported outbreaks [21–47], with descriptions and references collected in Table A1. Both simulations (even in this simple model) and real outbreaks are highly variable in size and duration, though a 2–3 month duration is common. The model produces “non-outbreaks”, where with several introduced cases, transmission does not take off sufficiently to lead to an outbreak. These, of course, also occur in real-world settings and are not written up as outbreaks, and so do not occur in the table (however, the pattern can be seen in public health reports. For example, there were seven measles cases reported in Ontario in 2017; five reported travel outside of Ontario in the time period seven to 21 days before rash onset: two to India, one to Pakistan, one within Canada, and one to Mexico [21]). Our model results are most similar to outbreaks of Donggang [22], Gothenburg [23], Edinburgh [24], and Waterloo [25]. For $80\%$ or higher coverage, our model produced similar outbreaks to the outbreaks of Stark County [26] and Saint John [27], assuming a moderate level of public health intervention, e.g., catch-up vaccination programs are accepted at a high rate.

## Discussion

We constructed a simple SEIR model to convey what measles outbreaks could look like in Canada under various public health responses and vaccination levels within a community. The parameter choices for our model were informed by historical data from previous measles outbreaks in Canada. Features of simulated outbreaks reflect previously-reported outbreaks, a number of which are described in Table A1.

Measles outbreaks are sometimes reported, even large ones, where reported vaccination coverage is high. This is likely due to the jurisdiction or level where it is feasible to report coverage not being reflective of one or more vulnerable (to measles) communities in that jurisdiction (for example, Brooklyn, NY as a whole, Lyon as a whole, in contrast to religious or other connected communities within those jurisdictions, with lower coverage, or large numbers of visitors (Disneyland outbreak)).

Of the outbreaks shown in Table A1 reported outbreak sizes range from under ten to over 100 and last 1–8 months. Interestingly, most outbreaks with community vaccination coverage at least $80\%$ have similar trajectories in terms of outbreak size and duration. We suspect that the lack of difference between outbreaks in communities with $>90\%$ coverage and 80–90% is due to public health interventions. In our model, outbreaks in communities with $\ge 90\%$ coverage last up to two months and do not tend to reach over 20 individuals. In the second scenario, we did not consider coverage below $55\%$, however, we believe our model may overestimate outbreak sizes at those lower vaccination coverage levels. Outbreaks might be limited by population structure (unequal mixing) along age, gender, school, religious institution or other aspects; reported outbreaks may include transmission in distinct, but interlinked, subgroups in a community. Our model does not explicitly account for the potentially unique interaction patterns of these communities. It is interesting to note that vaccination coverage does not predict outbreak duration (there is a wide range of durations across coverage levels). Many outbreaks in communities with low vaccination coverage have similar or shorter duration compared to communities with high vaccination coverage (see Table A1) which is also true in our model. Finally, there are many communities with intermediate coverage where outbreaks are comparable to our model results, depending on the strength of public health intervention.

Modelling is used for scenario projections and risk assessment in a range of infectious disease risks, including measles [4]. Measles outbreaks are a concern and are described in the literature. However, a lack of small but key pieces of contextual information limits what we can learn from reported outbreaks, and limits the use of models and even qualitative comparisons to better understand measles risk. Information about the vaccination coverage in the affected group before the outbreak, the approximate community size (or numbers exposed but not infected), and the nature, extent and timing of the public health response would be extremely helpful.

We modelled outbreaks that each take place in a single unified community; however, one outbreak in a fairly well-defined community could seed another. A string of 4–6 outbreaks with durations typical of those in our model could last a year or more, challenging elimination status, particularly if multiple introductions occur along with one or more chains of outbreaks. But our modelling suggests that unless a series of linked outbreaks or a large number of introductions (that cause outbreaks) to vulnerable communities occur, Canada is unlikely to lose elimination status.

Previous studies have modelled measles outbreaks [28–30] considering vaccination status and/or transmission dynamics. However, to our knowledge, none have included public health interventions such as contact tracing, PEP, and self isolation, which play an important role in outbreak management.

Age affects the risk of severe measles outcomes, and contact patterns affect transmission dynamics. However, without knowledge of the kind of community that is experiencing an outbreak we cannot model age and contact patterns (including age-related contacts). If the community were highly structured, transmission would probably be slower, and outbreaks smaller (though perhaps of longer duration) than shown here. However, simple transmission models are a mainstay of infectious disease scenario modelling because they are informative despite making simplifying assumptions.

We have not explicitly modelled introductions. The rate at which they occur will be be a product of the travel volumes to and from higher-incidence regions, the measles burden in those regions, the vaccination status of travellers and the contact between travellers and people with measles infections. The outbreak risk given an introduction depends (as we have modelled here) on the vaccination coverage in the affected community. It is hard to determine how many travel-related introductions will expose vulnerable communities. Finally, it is challenging to translate public health measures into a simple modelling framework, particularly one (such as ours) with compartments linked by rates of movement. This is a limitation especially for modelling preventive measures for unvaccinated individuals who were exposed (in the sense that they were in contact with an infectious individual), because not all susceptible individuals are exposed at the same time. More detailed agent-based or network models could readily be developed given further information about the communities likely to be affected (age, contact structure, likely policy or recommendation following exposure, etc). In any case, our approach to modelling interventions is approximate.

## Conclusions

This work may serve as one piece of a broader effort to understand the risks resulting from measles importation and to inform potential public health responses. Since the vast majority of measles cases in Canada can be traced to imported cases, this model serves as a last step in the chain of actions needed to bridge from global measles outbreaks to local scenarios within Canada. Given cases entering Canada, we are able to project what an outbreak may look like, helping to inform the public of the measles-related risk, quantify potential benefits of further encouraging vaccination uptake and informing the public of measles-related risks. To further aid public health agencies, we have an R Shiny web application (jmcn.shinyapps.io/measles-canada) to accompany this work which provides an interactive tool to model public health interventions in the model discussed here.

## Supplementary Information

Supplementary Material 1.

## Data Availability

Our simulation (including generated data) is fully reproducible with the code available at https://github.com/jmcnichol/measles-canada.

## Abbreviations

WHO: The World Health Organization
SEIR: Susceptible-Exposed-Infectious-Recovered
PEP: Post-exposure prophylaxis
MMR: Measles, Mumps, and Rubella
