# Supplementary material for: Measles in Canada: modelling outbreaks with variable vaccine coverage and interventions
Source: BMC Infect Dis. 2025 Feb 19;25:236. doi: 10.1186/s12879-025-10564-8 (PMC11841303; doi:10.1186/s12879-025-10564-8)
Supplement: Supplementary file 1 — Supplementary Material 1. [file 12879_2025_10564_MOESM1_ESM.pdf]

Appendix

Figures A1 and A2 show outbreak size and duration, respectively, for simulations in a community of size 8000. Figure A3 shows the sensitivity of parameters q_i, q_s, q_{pep} and v, in relation to outbreak size and duration.

Figure A4 shows outbreak sizes over 100 simulations in population size of 1000 at vaccination coverages of 0.7 and 0.9 given the number of initial infections imported.

Figure A5 shows the outbreak size distributions and durations in an alternative model set-up, in which there is no infectivity in the E class, and all infectious individuals are in the I class. Individuals have rash 4 days into their infectious period. This results in parameter changes that are required for the model to be reasonable. The rate of transition from E to I is 1/(8 days) instead of 1/(12 days). The rate of removal from I to the recovered class R is 1/(8 days) instead of 1/(4 days). Not knowing that they are infectious before rash, individuals do not isolate as soon, so $q_i = 0.076, q_{pep} = 0.90, q_s = 0.054, c = 0, v = 0.003, \gamma = 1/8$ and $k = 1/8$. Finally, the transmission parameter β was adjusted to keep the basic reproduction number consistent, using the equation $R_0 = \frac{c\beta S_0(\gamma+q_i)+k\beta S_0}{(k+q_{pep})(\gamma+q_i)}$. Let the original parameters be denoted with a superscript o (for example c^o) and the new ones with an n (for example c^n). We set β^n such that

$$\frac{c^n\beta^n S_0(\gamma^n + q_i^n) + k^n\beta S_0}{(k^n + q_{pep}^n)(\gamma^n + q_i^n)} = \frac{c^o\beta^o S_0(\gamma^o + q_i^o) + k^o c^o\beta^o S_0}{(k^o + q_{pep}^o)(\gamma^o + q_i^o)}.$$

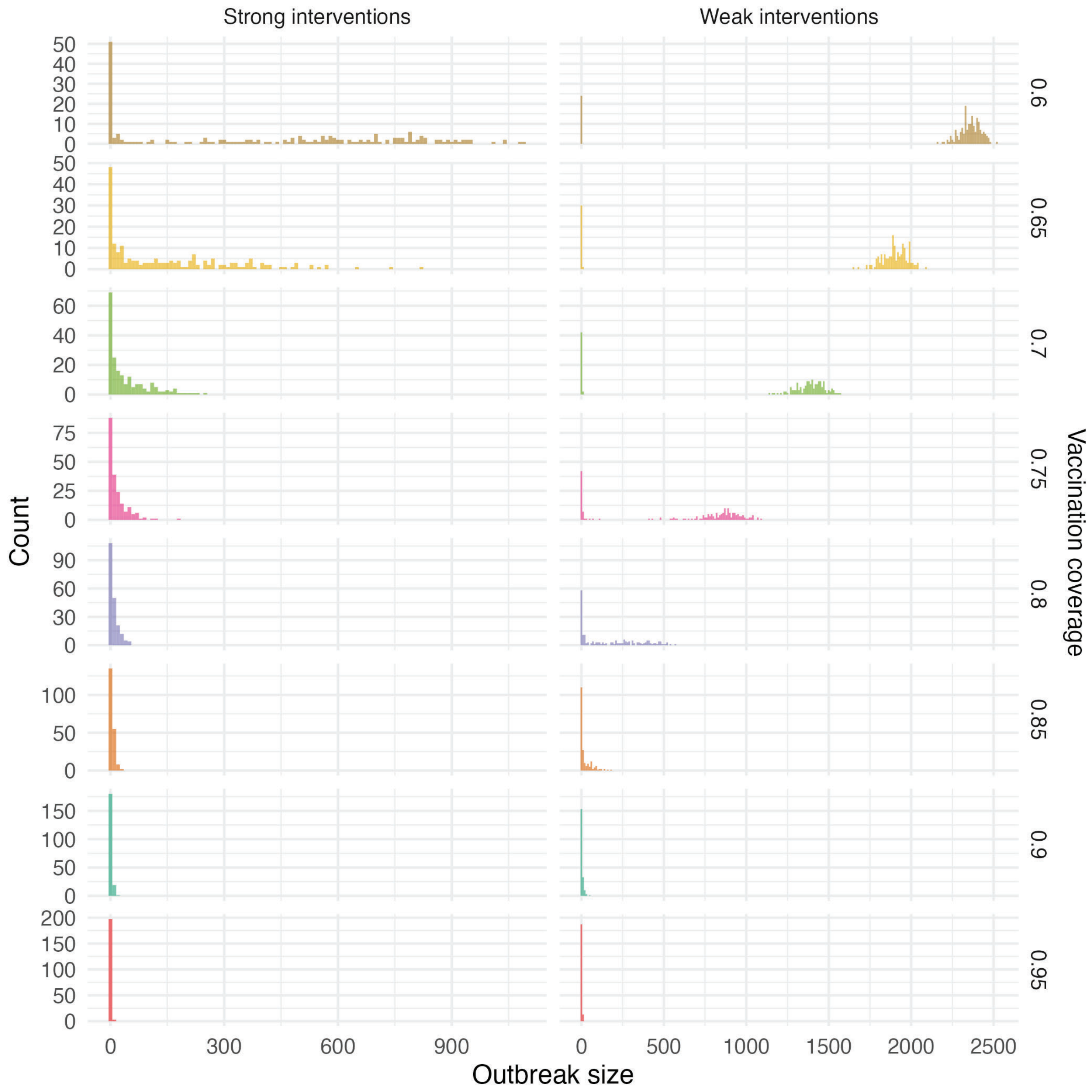

Figure A1: Outbreak sizes for 100 simulations of a population of size 8000 with strong and weak interventions for various levels of vaccination coverage.

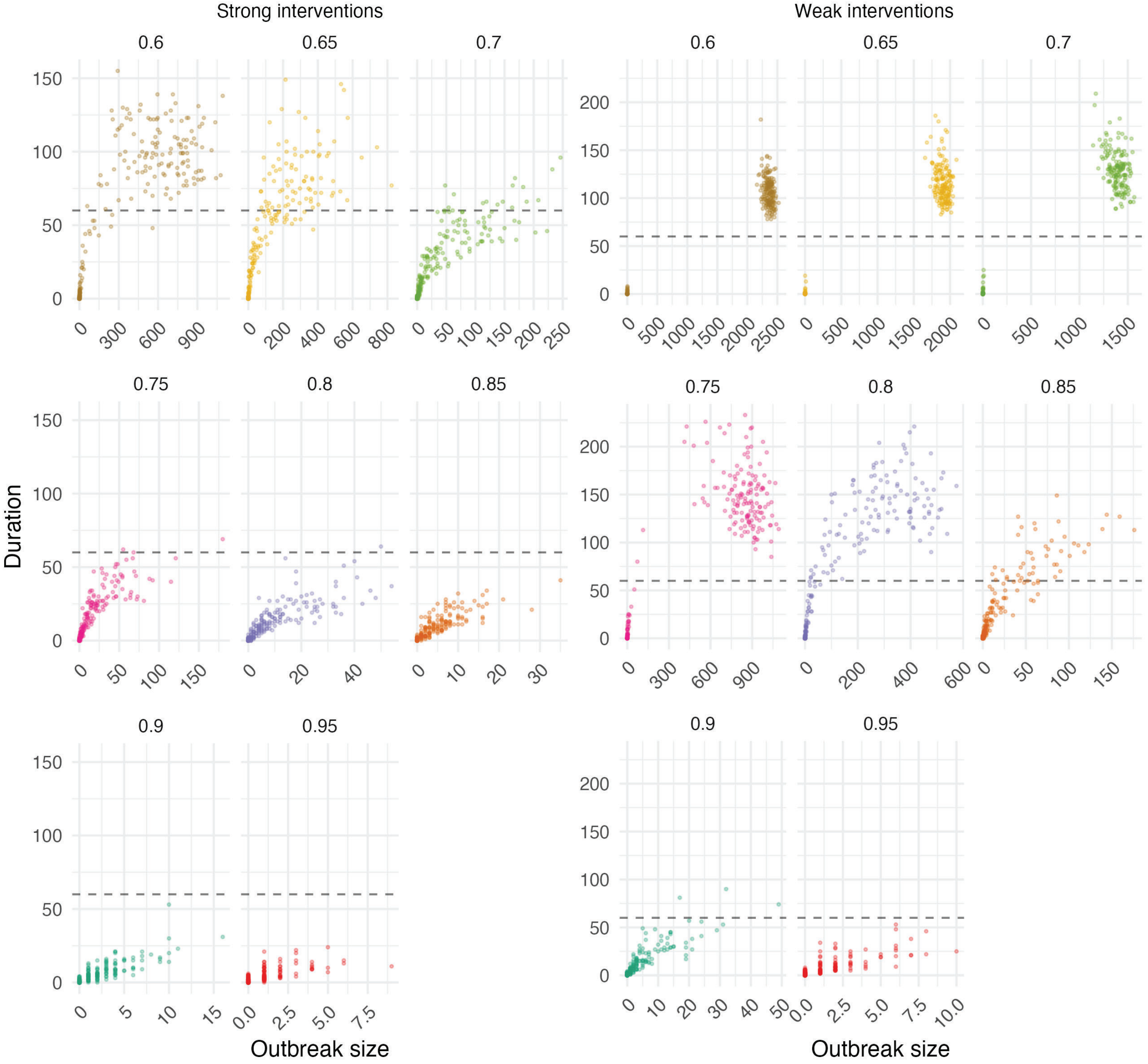

Figure A2: Duration (in days) versus outbreak size over 100 simulations of a population of size 8000 with strong and weak interventions for various levels of vaccination coverage. Grey dashed line represents 60 days.

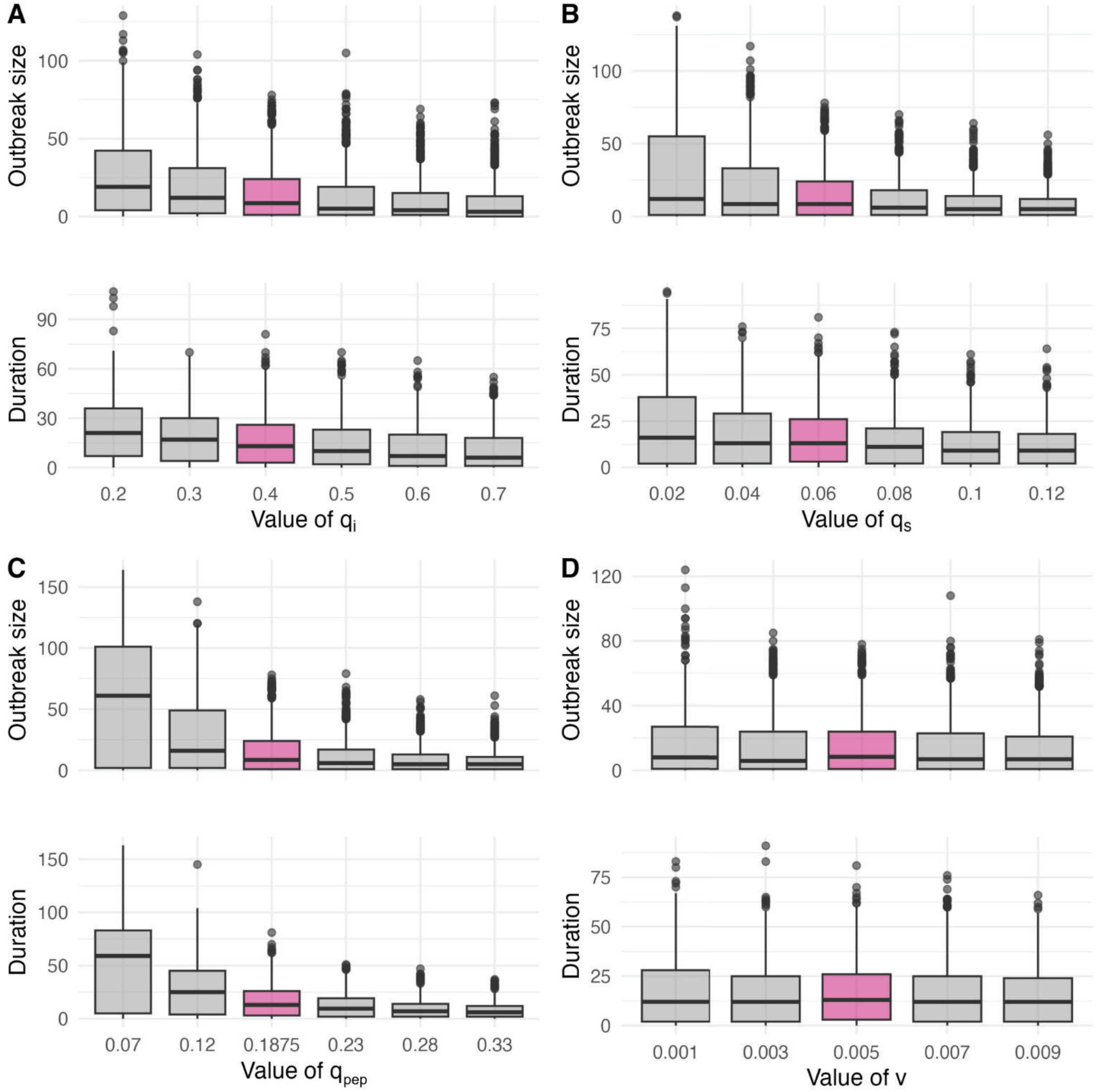

Figure A3: Sensitivity of parameters (a) q_i, (b) q_s, (c) q_{pep} and (d) v, in relation to outbreak size (top of each panel) and duration (bottom of each panel). We performed 1000 simulations (with a vaccination coverage of 0.75 and population size of 1000) of our model varying each of the four parameters independently. Pink indicates the parameter value that was used in our simulations, i.e., the default value of the parameter under "strong interventions" (described in Table 1).

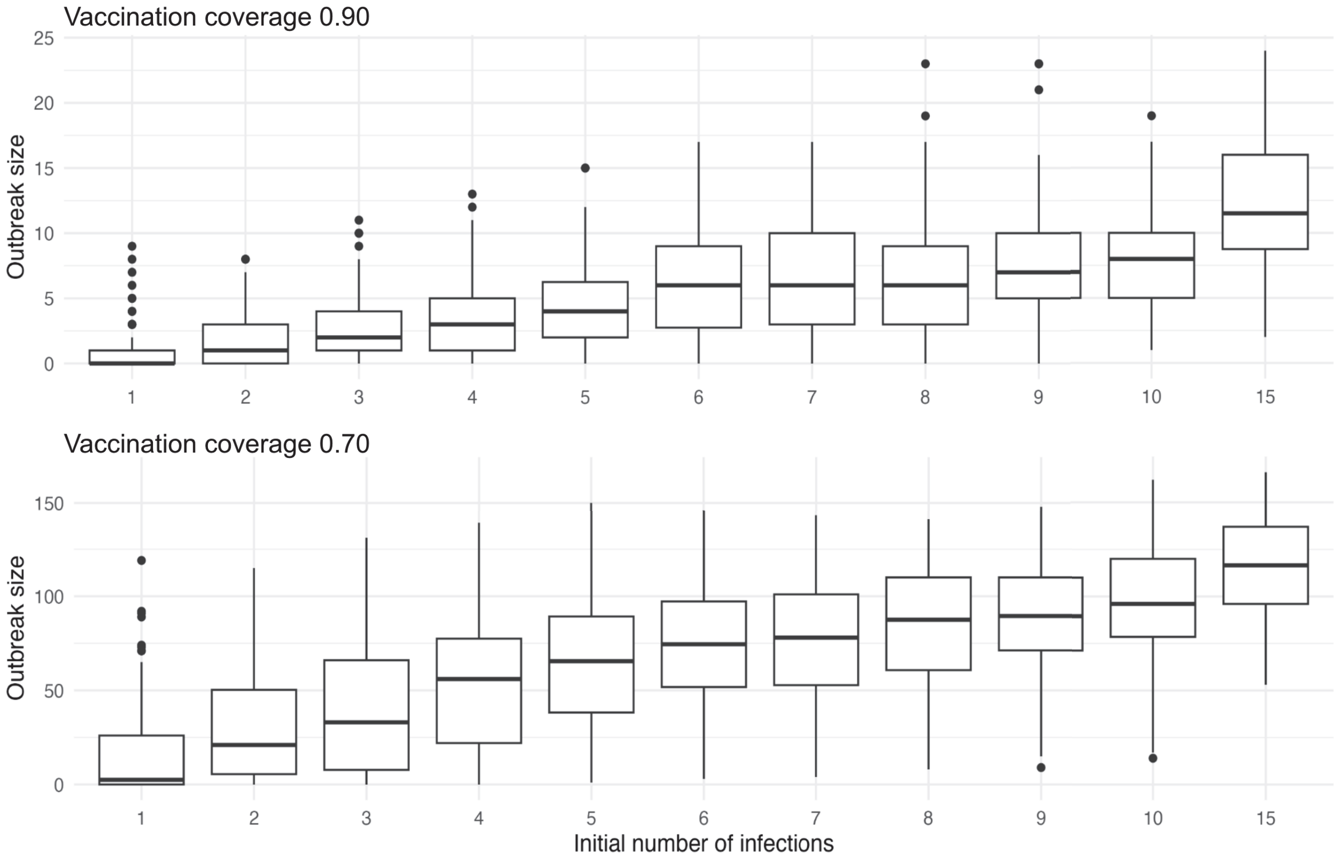

Figure A4: Variation in outbreak size given the initial number of infections imported into comminutes with 90% vaccination coverage and with 70% vaccination coverage. 100 simulations were performed with a population size of 1000.

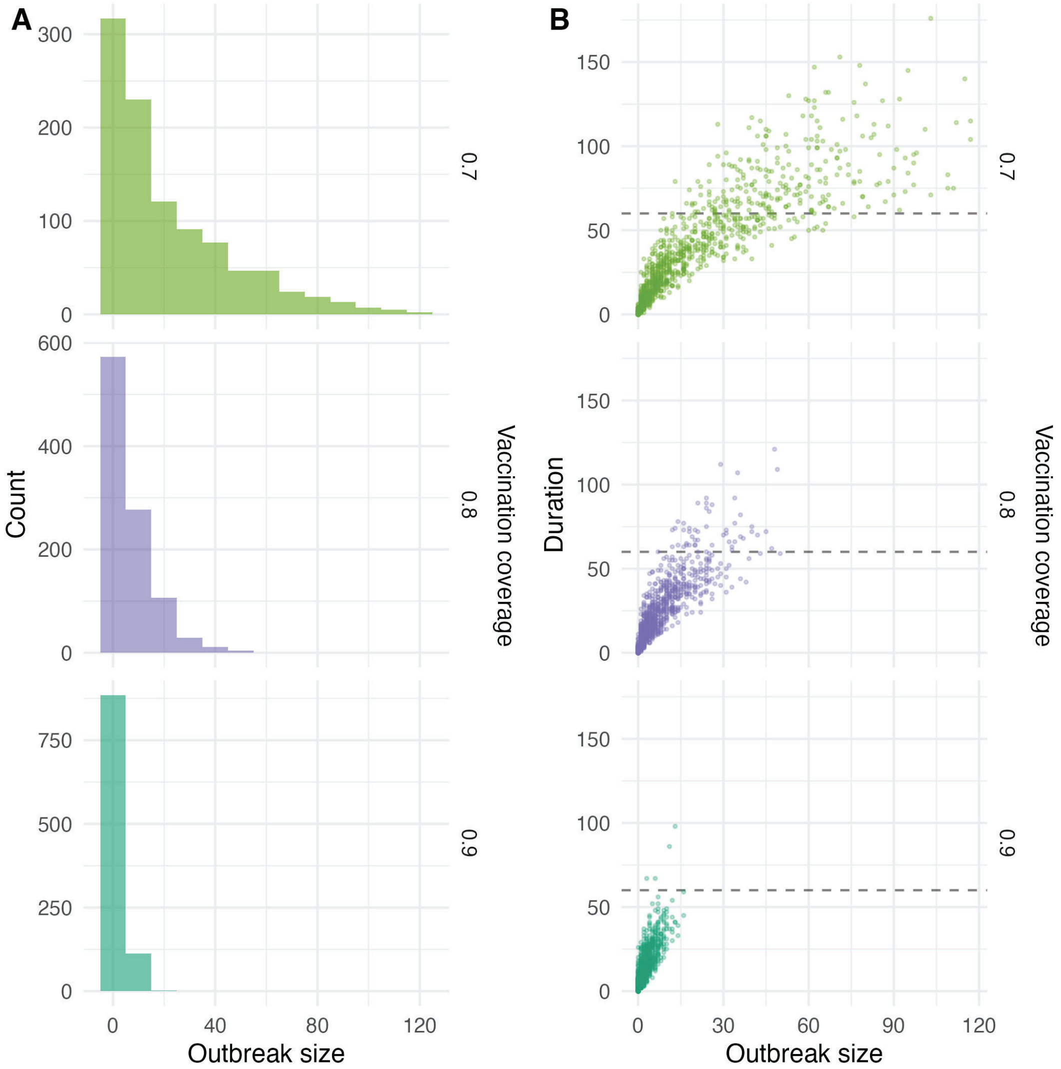

Figure A5: Outbreak size distributions (a) and durations (b) in an alternative model set-up, in which there is no infectivity in the E class, and all infectious individuals are in the I class. We performed 1000 simulations at three vaccination coverage levels for a population size of 1000.

Table A1: Information on select outbreaks in comparable regions to those across Canada, by year. "Community type" denotes the type of community that measles began to spread in after importation. Vaccination coverage is the proportion (of the most relevant population for which coverage is reported) with at least one dose of measles containing vaccine estimated average among all age groups in the community unless otherwise specified. Durations are approximate. "Public health measures" indicates which initiatives the PH agency in that jurisdiction implemented upon declaration of the outbreak. Vaccination campaign includes community and/or in-school vaccination clinics and promotion of up to date vaccination status, and vaccination of susceptible individuals. Public health advisories include promotion of measles-containing vaccine, awareness of measles outbreak and/or cases, and community education.

**Year**	**Location**	**Community type**	**Cases**	**Vaccination coverage**	**Duration**	**Public health measures**
1999	The Netherlands	Religious	213	7% [31]	11 months	monitoring, public health advisories
2006	Duisburg, Germany	General pop.	614 (city), 53 (school), 78 (school and households)	90% (school) [32]	5 months	vaccination campaigns, public health advisories

continues on next page

2007	Antwerp, Belgium	Religious	137 [33]	77% [34]	10 months	vaccination campaigns, active case finding and contact tracing, public health advisories
2008	Salzburg, Austria	Religious	233	34% [35]	5 months	vaccination campaigns, school closures, exclusion of unvaccinated from schools and public places, public health advisories
2009	Waterloo, Ontario	Unknown	7	>90% [25]	6 weeks	contact tracing, high compliance with self-isolation [25], vaccination campaigns, public health advisories

continues on next page

2010	Zaka, Masvingo, Zimbabwe	Religious	126	75% in Zaka; suspected to be lower in affected community [36]	4 months	vaccination campaigns, public health advisories, environmental health technicians to enforce vaccination in religious communities
2010	Lyon, France	General pop.	407	88% [37]	18 months	contact tracing, vaccination campaigns, PEP, virological and immunological testing for health care workers and patients, public health advisories

continues on next page

2011	Berlin, Germany	Religious/ school	73	49% [38]	4 months	school exclusions, vaccination campaigns, public health advisories, rapid laboratory diagnosis
2011	Ghent, Belgium	Religious/ school/ daycare	65	<50% in community, 0% in daycare [39]	2 months	isolation measures and exclusion of unvaccinated students, vaccination campaigns
2011	Hennepin County, Minnesota, USA	Emergency housing shelter	21	54% [40]	2.5 months	case follow-up, voluntary isolation and quarantine, vaccination campaign including early vaccination for non-immune shelter residents, public health advisories

continues on next page

2013	Lethbridge, Alberta	Religious	42 [41]	86% [42]	6 weeks	vaccination campaigns, dedicated measles hotline and assessment centre, quarantine and exclusion measures
2014	Chilliwack, BC	Religious	433	Unknown, low vaccination acceptance among this community [43]	2 months	travel restrictions, closure of affected school, PEP
2015	Disneyland, California, USA	Theme park	131 [44]	92% for California children [45]	3 months	contact tracing, vaccination campaign, travel history monitoring, public health advisories

continues on next page

2016	Edinburgh, UK	University	18	93% nationally but many university students were either unvaccinated or unsure of their vaccination status [24]	3 months	contact tracing, isolation of suspected and confirmed cases, vaccination campaigns, public health advisories, exclusion of high-risk unvaccinated contacts from educational settings
2017	Gothenburg, Sweden	Health care centre	28	95% [23]	1 month	large-scale contact tracing, PEP, vaccination campaigns
2017	Hennepin County, Minnesota, USA	Ethnic	65	35% [11]	1 month	Case follow-up, vaccination campaigns, PEP, public health advisories

continues on next page

2017	Sicily, Italy	Unknown	57	73% [46]	7 months	contact tracing, immediate isolation of suspected cases, vaccination campaign, public health advisory
2019	Donggang, Zhoushan Islands, China	Unknown	28	Unknown. Zhoushan Islands protective antibody rate of 46% [22]	2 months	Contact tracing, isolation of close contacts, vaccination campaigns, public awareness campaigns
2019	Brooklyn, New York, USA	Religious	649	79.5% (Williamsburg overall) [47]	9.5 months	Vaccination campaigns, exclusion of unvaccinated children from schools and childcare programs, extensive contact tracing, public health advisories

continues on next page

2019	Saint John, New Brunswick	School	12 [27]	87% [48]	5 weeks	Vaccination campaigns, public health advisories
2022	Stark County, Ohio, USA	Unknown	73	>80% [26]	3 months	Vaccination campaigns, public health advisories
